# Predicting Equilibration Dynamics of Polymer Confined at the Nanoscale via Material Time

**DOI:** 10.1021/acs.jpclett.5c00945

**Published:** 2025-05-14

**Authors:** Katarzyna Chat, Ewa Sikora, Karolina Adrjanowicz

**Affiliations:** † Institute of Nuclear Physics, 113066 Polish Academy of Sciences, 31-342 Krakow, Poland; ‡ Institute of Physics, 49569 University of Silesia, ul. 75 Pulku Piechoty 1, 41-500 Chorzow, Poland

## Abstract

Nonequilibrium phenomena are important in determining the dynamics of polymers confined at the nanoscale level. Growing experimental evidence demonstrates that nanoscale confinement giving rise to enhanced molecular mobility and deviation from the bulk behavior can be eliminated with time via very small density/volume changes that result from molecular rearrangements toward a more stable, i.e. less energetic, state. Remarkably, a similar effect is commonly observed upon physical aging of glasses. Here, we demonstrate that equilibration phenomena in nanopore confinement reveal the same fundamental features as the out-of-equilibrium response of (bulk) glasses subjected to various thermal histories. Following the concept of Narayanaswamy’s single-parameter aging, we describe the equilibration of polymer dynamics at the nanoscale. The collected data show that the material-time concept is also valid in nanopore-confinement. Thus, it is possible to predict the confined polymer response to a temperature jump directly from the knowledge of a single relaxation curve.

## Graphical abstract

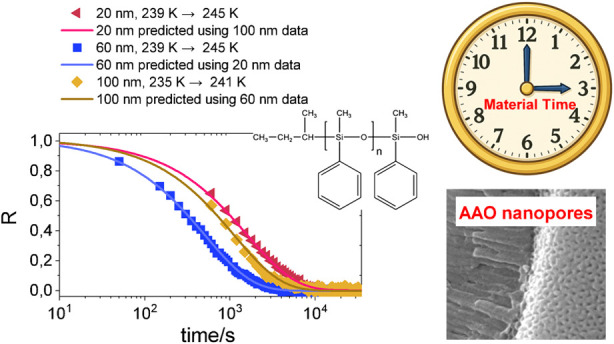

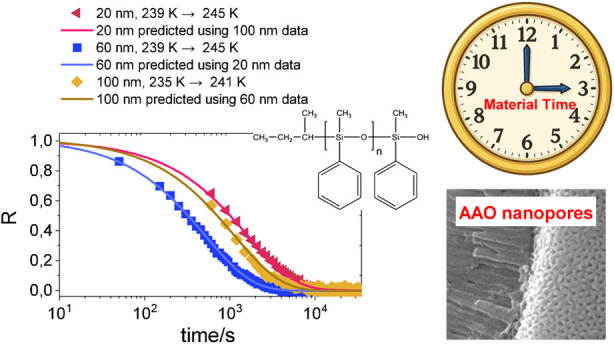

Polymer materials confined at the nanoscale are highly metastable forms of matter.^1^ The long-lived metastability of such systems comes mainly from the processing conditions, which are much faster than the time needed for structural rearrangement. As a result, spatially constrained polymer chains freeze in far-from-equilibrium conformations, bringing about dramatic changes in dynamics, such as reduced viscosity or enhanced mobility.^2–4^ However, deviations from bulk behavior observed at the nanoscale have a limited lifetime, and it is possible to recover some of the bulk-like properties by annealing for a sufficiently long time.^5–10^

Out-of-equilibrium phenomena observed for soft matter systems confined at the nanoscale level are of paramount importance for emerging applications in nanotechnology, giving access to novel functional materials such as polymer-based nanometer-size coatings or drug delivery systems. Their functionality and performance are highly related to the lifetime of the deviations from bulk behavior and conditions that would freeze the molecular system in the desired conformation for a time scale much longer than those of interest for technological applications. Description of the structural recovery under geometrical confinement is also essential for building a complete picture of the glass transition dynamics. Hence, knowing how to predict such time-dependent changes is of great scientific and practical importance.^3,11^

Here, we describe the equilibration kinetics of a polymer confined at the nanoscale level. For that, we have used the “material time” concept proposed many years ago by Narayanaswamy, an engineer from Ford Motor Company, who looked for a model to predict the physical properties of macroscopic glassy materials subjected to complex temperature–time histories occurring upon industrial processing. This led to the famous Tool–Narayanaswamy (TN) formalism, which captures the essential complex behavior of the structural recovery process, such as nonexponentially in time and nonlinearity in temperature variation in terms of a single parameter, so-called “material time”.^12–15^ The clever output of Narayanaswamy is the assumption that any aging system has an “inner clock” that measures the material time. The material time quantifies how fast dynamic processes in an aging system take place at a rate that changes as the structure of glass ages.^14^ Although the fundamental meaning of the material time remains elusive, the TN formalism (and its further modifications) is unquestionably successful in describing the physical aging of bulk glasses subjected to various thermal protocols.^11,16–23^ Notably, the existence of a material time—though the entire concept has been known for more than half of a century—has been validated in experimental studies only recently.^24,25^

Description of the equilibration phenomena in terms of the TN formalism and the existence of a material time have never been tested under nanopore confinement. We do this by performing time-dependent experiments on a modeled polymer glass-former embedded into cylindrical nanochannels of different sizes and subjected to various thermal histories. It is worth acknowledging, however, research works on physical aging on silica nanoparticles successfully described using the Tool–Narayanaswamy–Moynihan model.^26,27^ Our experiments are based on monitoring evolution in the dielectric response of the tested polymer embedded inside cylindrical alumina nanopores after temperature jumps of different magnitudes. A similar experimental approach to track equilibration kinetics via dielectric spectroscopy has been reported by Napolitano et al. for thin polymer films^6,28^ and by Kardasis et al. for nanopore-confined polymers of different architecture.^29^ In this study, we have used a commercially available dielectric spectrometer with a temperature control system, ensuring temperature stability up to 0.1 K. The α-relaxation time is determined based on dielectric data and then used to analyze equilibration kinetics. The tested sample is poly­(phenylmethylsiloxane), our modeled polymer glass-former, with molecular weight *M*_*w*_ = 2530 g/mol and polydispersity index 1.4 (Polymer Source, Canada). It will be termed PMPS 2.5k in this paper. Geometrical nanoconfinement was introduced by utilizing commercially available anodized aluminum oxide (AAO) nanoporous membranes characterized by uniform arrays of unidirectional and non-cross-linked cylindrical channels (Inredox, USA). Materials and Methods in the Supporting Information details the experimental methods and data treatment protocols.

A viscous system kinetically freezes and falls out of equilibrium when the relaxation time is so long that it cannot reach equilibrium within an experimentally given time scale.^30^ For bulk polymer this happens when the time scale of the structural relaxation reaches 1 s.^31^ In the glassy state, there is no direct access to the α-relaxation dynamics. Thus, the only parameter measured upon physical aging of bulk glass is the dielectric permittivity on the high-frequency flank of the α-relaxation peak (see Figure 1a). In contrast, under nanopore-confinement kinetic arrest of the interfacial layer, i.e., a fraction of polymer chains strongly interacting with the pore walls,^32–39^ results in nonequilibrium behavior observed for segmental dynamics even 6–8 decades faster than in bulk (Figure 1b). In such a case, the maximum of α-relaxation is constantly seen in the experimentally accessible frequency window (see Figure 1c and Figure 1d). This is an invaluable asset because equilibration kinetics can be monitored directly by tracking changes in the α-relaxation time. When the equilibration process is completed, the confined material recovers τ_α_ characteristics for the bulk material. Nonequilibrium phenomena studied in the present work is adsorption/desorption to/from the surface of the pores. This is a different phenomenon from the physical aging of bulk glasses. Nevertheless, in the following we show that even such different systems exhibit comparable signatures.

**Figure 1:**
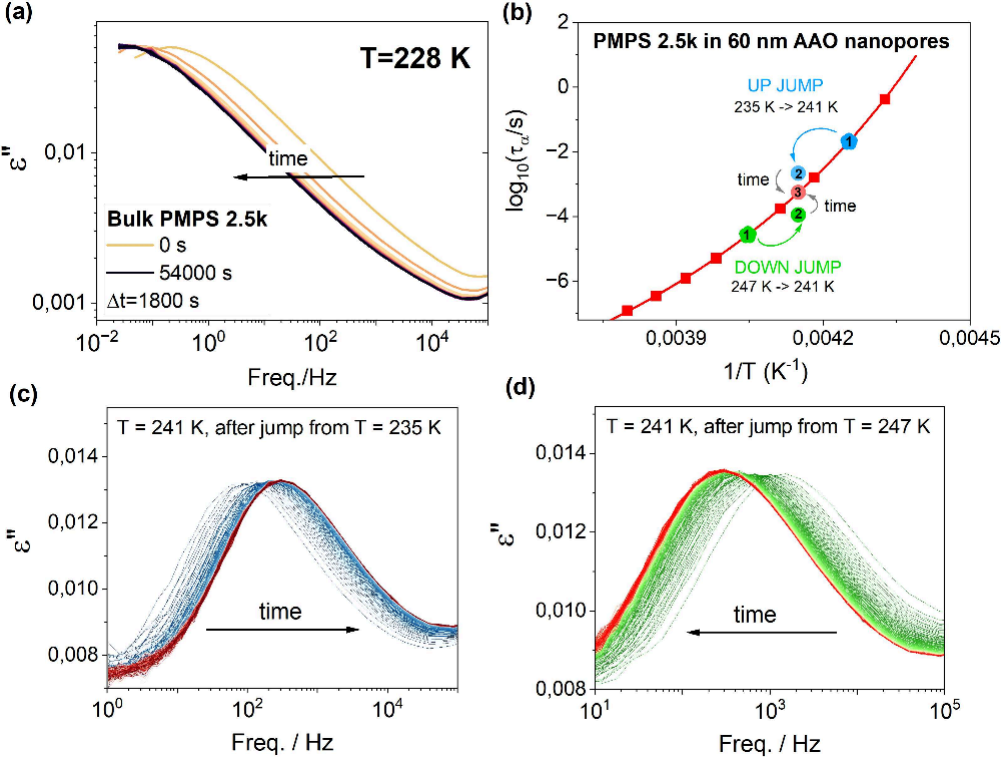
Differences in the equilibration phenomena in bulk and nanopore-confined polymer glass-former followed by dielectric spectroscopy. (a) Dielectric loss spectra for bulk PMPS 2.5k aged just below *T*_g_. (b) Temperature dependence of the α-relaxation time for the bulk polymer (red squares) plotted together with the experimental data measured in 60 nm pores in the temperature region where out-of-equilibrium phenomena are observed. Changes in the dielectric loss spectra as a function of annealing time recorded in 60 nm pores at 241 K after 6 K (c) up and (d) down temperature jumps.

Narayanaswamy’s material-time description approaches the aging analysis not in the actual experimental time *t*, but a dimensionless (“reduced”) material time, *t*, defined using internal clock rate:

1 $$d\mathrm{t̃}=\gamma \left(t\right)dt\Leftrightarrow \mathrm{t̃}=\int_{t}^{0}\gamma \left(t\right)dt$$

During physical aging the clock rate, γ­(*t*), “ticks” at the same rate as the glass structure changes, and it is linked to *X*(*t*), i.e., the property monitored upon the equilibration process at a given temperature *T*. The normalized relaxation function of *X*(*t*) is described as

2 $$R\left(t\right)=\frac{X\left(t\right)-X_{eq}\left(T\right)}{\Delta X\left(0\right)}$$

where Δ*X*(0) is the total change of the followed property from the equilibration start to the equilibration end while *X*_eq_(*T*) is the equilibrium value of *X*. The limiting values of *R*(*t*) are 1 (*t* = 0) and 0 (*t* → *∞*). Since the TN formalism assumes that *R* is a unique function of material time, all collected normalized relaxation functions *R*(*t*) should collapse onto a single curve when plotted versus reduced (material) time, *R* (t̃) . In this way, the TN formalism predicts that for any temperature jump, the system relaxes toward equilibrium following the same function as the material time.^17^

In agreement with the TN formalism, the rate of progress toward equilibrium is governed by the current value of the relaxation time:

3 $$\gamma \left(t\right)=\frac{1}{\tau \left(t\right)}$$

Since in nanopore confinement we have a very unique opportunity to monitor the actual changes in the structural dynamics upon the equilibration process, the clock rate is approached right from the experimental data. In the following, we do it via time-dependent characteristic time, τ­(*t*), more commonly denoted as the aging (or annealing) time constant. The time-dependent characteristic time can be determined from the analysis of the response function *R*(*t*) using a relaxation function similar to stretched exponential and interpreted as an averaged relaxation time representing the entire aging process until time *t*.^17,40–42^ For bulk glasses, this is far more complicated because there is no direct access to the relaxation time or the clock rate. In such a case, one relies only on the temperature and structure shift factors.

Before testing the material time concept under confinement, we need to demonstrate that the equilibration phenomena observed in nanopores reveal the same characteristic features as the physical aging of bulk glasses. In agreement with Kovacs and McKenna’s works, the fundamental signatures of structural recovery are (i) intrinsic isotherms, (ii) asymmetry approach, and (iii) memory effects.^11,20,23,43,44^ The first denotes that for a series of temperature jump experiments from an equilibrium state located close to the glass transition temperature (*T*_g_) to a series of temperatures below *T*_g_, the structural recovery curves shift rapidly toward longer times with decreasing temperature and the calculated equilibration times increase exponentially. Figure 2a confirms intrinsic isotherms for the tested polymer confined in 60 nm alumina templates. The next experiment is the “asymmetry of approach”, revealing the inherent nonlinearity in temperature and structure of the recovery process. In this case, one compares the equilibration kinetics at the same temperature but approached following two different temperature jumps of the same depth, ±Δ*T*. A typical observation reported for numerous bulk-glass-forming systems is that these two responses are not identical. Shorter equilibration time is reported for down jumps.^12,18,20,45^ Figure 2b illustrates the results of the asymmetry approach experiment in 60 nm pores. In nanoconfinement, down jumps result in faster and more stretched responses than up jumps. The results of analogous experiments performed at a fixed temperature (245 K) as a function of the pore size are presented in Figure 2c. The recovery of the bulk-like dynamics slows down with decreasing pore size, but it remains valid that the down jump equilibrates faster. This is because the down jump starts from the condition of the surplus volume and faster mobility compared to the equilibrium response. On the other hand, for an up jump, the initial state is characterized by a deficit of volume, which means that the molecular mobility is reduced compared to that in the equilibrium bulk state. The equilibration rates in confinement are always at least 6–7 decades slower compared to those in τ_α_ (see the Supporting Information for further analysis).

**Figure 2:**
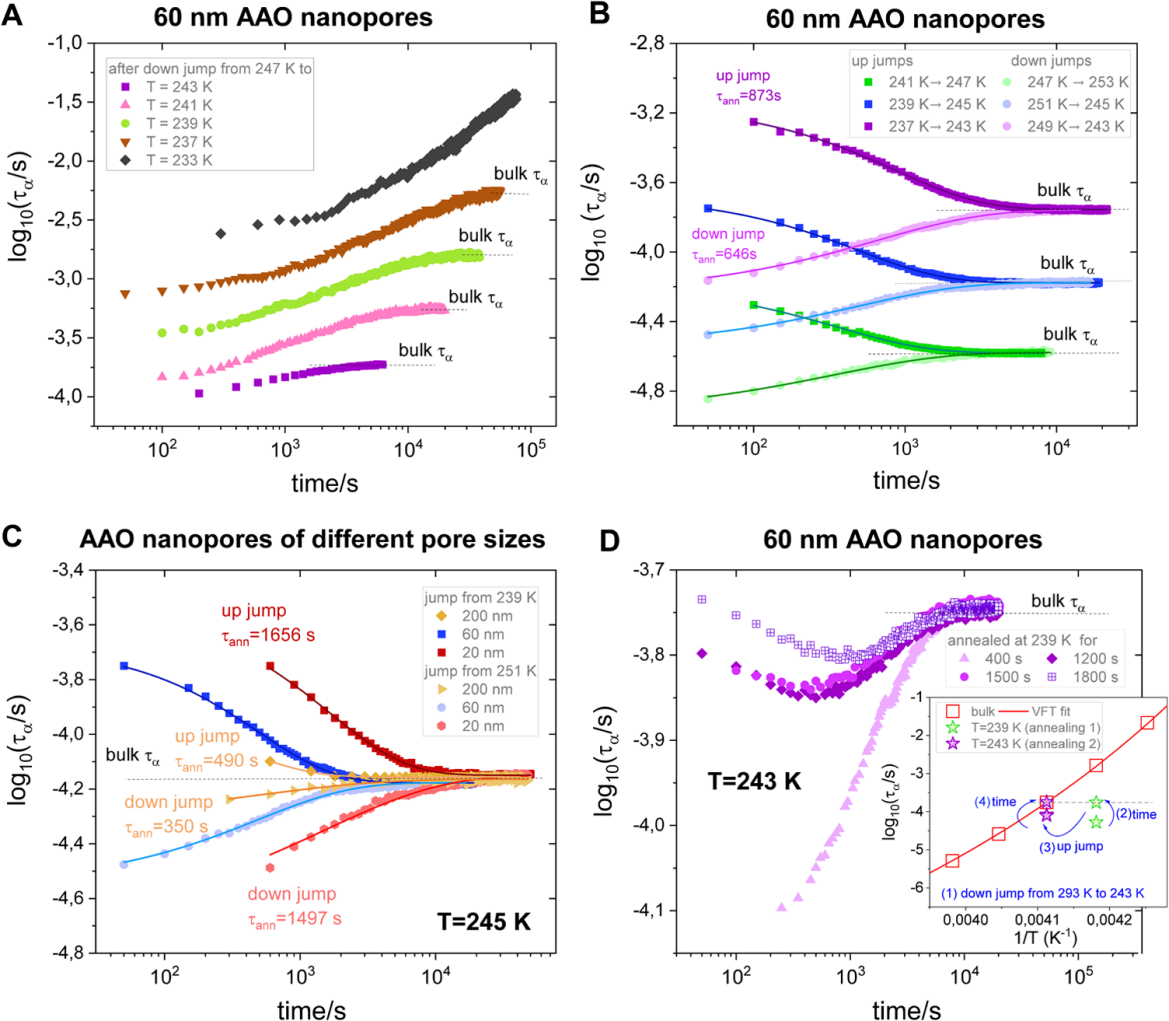
Three fundamental features of the structural recovery revealed for polymer glass-former under confinement. Changes in α-relaxation time as a function of annealing time for PMPS 2.5k confined in 60 nm pores recorded (a) at five different temperatures after a series of down jumps from the same starting temperature, *T* = 247 K and (b) after up and down jumps to three selected annealing temperatures (243, 245, and 247 K). (c) Variation of α-relaxation time as a function of annealing time after up and down jumps to *T* = 245 K collected in pores of different sizes. In each case, the jump magnitude is the same, Δ*T* = 6 K. Solid lines are fits of the experimental data to a function similar to stretched exponential. (d) Changes of τ_α_(*t*) in 60 nm AAO nanopores at 243 K after initial preannealing carried out at lower temperature (239 K) for different amounts of time. The schematic of the thermal history for the memory type of experiment is presented in the inset.

The “memory effect” aimed to show that structural recovery response in polymers depends on the path history and that the dynamics show a broad distribution of the relaxation functions. In this case, the out-of-equilibrium system is subjected to a two-step temperature history. In the first step, it is allowed to partially and isothermally recover at the initial temperature *T*_1_, *T*_1_ < *T*_g_. Then, it is heated to a higher temperature *T*_2_, *T*_1_ < *T*_2_ < *T*_g_. The partial recovery time at *T*_1_ and the starting condition for the second jump are chosen to ensure that the departure from equilibrium at *T*_2_ is close to zero. However, typically, after jumping from *T*_1_ to *T*_2_ the glassy material does not immediately reach the equilibrium but nonmonotonically evolves with time. The inset in Figure 2d portrays our experimental approach to test the “memory effect” in confinement. After a down jump from 293 to 239 K, time-dependent measurements are performed. The initial and final values of α-relaxation time recorded at 239 K for 1800 s annealing are included in the inset in Figure 2d. As can be seen, τ_α_ increases with equilibration time, and at some point, it reaches the same value as the bulk polymer at 243 K. Once this happens, an up jump- in temperature is performed to 243 K, and again, changes in the dielectric response of the confined sample are recorded as a function of time. The results presented in Figure 2d (main graph) show that the nanopore-confined polymer did not instantaneously reach equilibrium after a second jump. Instead, it “remembers” prior history and approaches the bulk value of τ_α_ by passing through a maximum with the magnitude that depends on the initial annealing time at 239 K. This is a manifestation of the nonexponentiality of the relaxation process under isothermal conditions of the second step. In this way, we have confirmed that equilibration phenomena observed in nanopore-confinement reveal the same hallmarks as the physical aging of bulk glasses.

Normalized relaxation curves for the tested polymer confined in nanopores are shown in Figure 3. Figure 3a collects the results in 60 nm pores for various down jumps, while Figure 3b shows *R*(*t*) for 6 K up/down jumps in pores of different sizes (20, 60, and 100 nm). In the Supporting Information, we also show normalized *R*(*t*) dependence for up and down temperature jumps in 60 nm pores. The TN formalism predicts that for all temperature jumps—small or large, up or down—the normalized relaxation function is a unique function of the material time.^17^ Therefore, all *R*(*t*) dependences were converted to *R* (t̃) using eq 1, with the clock rates obtained via time-dependent characteristic time (please see the Supporting Information for details). The results are presented in Figure 3c,d. Transformation from laboratory time to material time collapses all of the data onto a single curve, irrespective of the jump magnitude or pore size. This is the first experimental evidence that the material-time formalism can also be applied to nanoscale confinement.

**Figure 3:**
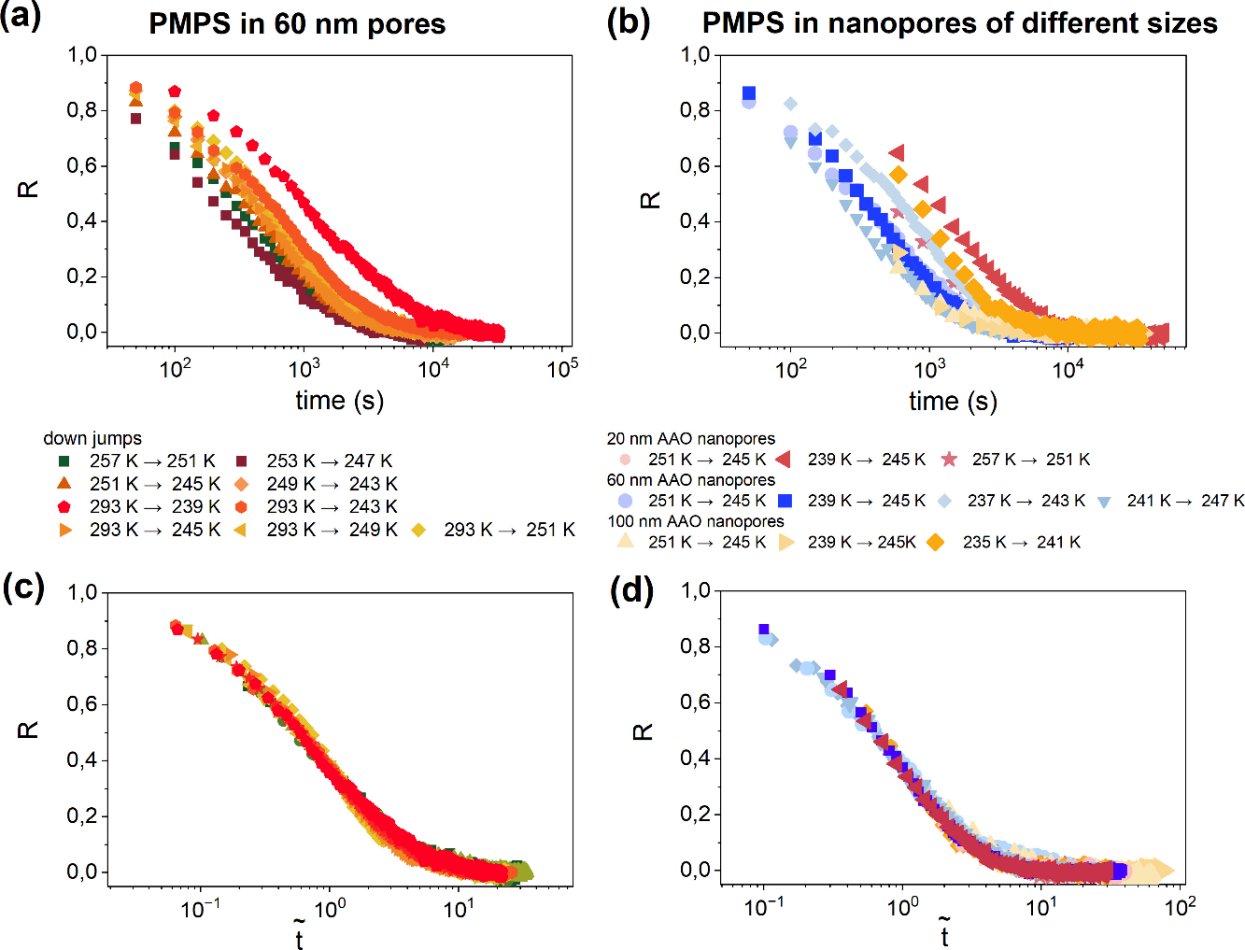
Traditional way of demonstrating TN material time concept applied for a nanopore-confined polymer glass-former, PMPS 2.5k. Normalized relaxation function for various down jumps plotted versus laboratory time (a and b) and reduced time, *t̃* (c and d). Results for 60 nm pores are shown in (a) and (c). (b) and (d) refer to normalized response functions collected in nanopores of different sizes.

Single-parameter-aging (SPA) is a simplified version of the TN formalism developed to describe nonlinear physical aging of bulk glasses.^14,17–19,22,24^ One of the most exciting aspects of SPA is the ability to predict a relaxation curve from one jump by using a relaxation curve from another jump. Herein, we test SPA to describe the equilibration phenomena in nanopores. The prediction needs as an input two temperature jumps A and B, having at some time *t*_A_^*^ and *t*_B_^*^ the same values of the normalized relaxation functions, *R* = *R*_A_ = *R*_B_. In the generalized version of the test, the jumps are not required to have the same magnitudes or final temperatures. The idea behind the prediction is that according to the TN formalism, *R*(*t*) is a unique function of the material time *t̃*, the same as its first derivative with respect to time. Thus, for the incremental changes of d*t*_A_^*^ and d*t*_B_^*^ this leads to identical changes of d*R*_A_ and d*R*_B_ and imposes the same condition for the material time $d\mathrm{t̃}\left(t_{A}^{*}\right)$ = $d\mathrm{t̃}\left(t_{B}^{*}\right)$ . On this basis, one gets the following^17,19^

4 $$t_{B}^{*}=\frac{\gamma_{eq,A}}{\gamma_{eq,B}}\int_{0}^{t_{A}^{*}}\exp\left(a\frac{X_{A}\left(0\right)-X_{B}\left(0\right)}{X_{eq}}R_{A}\left(t_{A}^{*}\right)\right)dt_{A}^{*}$$

which determines how a specific time step A can be transformed to the corresponding time step B knowing (1) the total change of the followed property for each jump, (2) the normalized response of jump A as a function of time, (3) the equilibrium value of the measured property, (4) a constant, and (5) equilibrium clock rates for each jump.

The results of the generalized SPA tests, expressed via eq 4, are demonstrated in Figure 4. Solid lines are predictions,while the experimental data are given as symbols. Figure 4a shows the prediction of one down jump from another down jump in 60 nm pores. The jump magnitudes are either Δ*T* = 3 or 6 K. The prediction collapses almost exactly with the data. For additional results demonstrating prediction for pairs of up and down jumps ending at the same equilibration temperature, please see the Supporting Information. Results obtained for large *T*-jumps are shown in Figure 4b. The prediction follows the data with a high accuracy, though some slight deviation is observed when a small temperature jump is used to predict a large temperature jump. In general, the SPA formalism breaks down for large jumps, as it is based on first-order Taylor expansion.^17,19^ In the case of bulk glasses, deviations between data and predictions was alternatively rationalized by molecular mechanisms other than those involved in smaller jumps.^48^ However, the kinetics of adsorption in the conditions of confinement employed in the present work is controlled by α-relaxation, as demonstrated by the ability to rescale all data within the SPA approach. In thin films, some studies also indicate that the absorption kinetics is controlled by the α-relaxation.^28,49^

**Figure 4:**
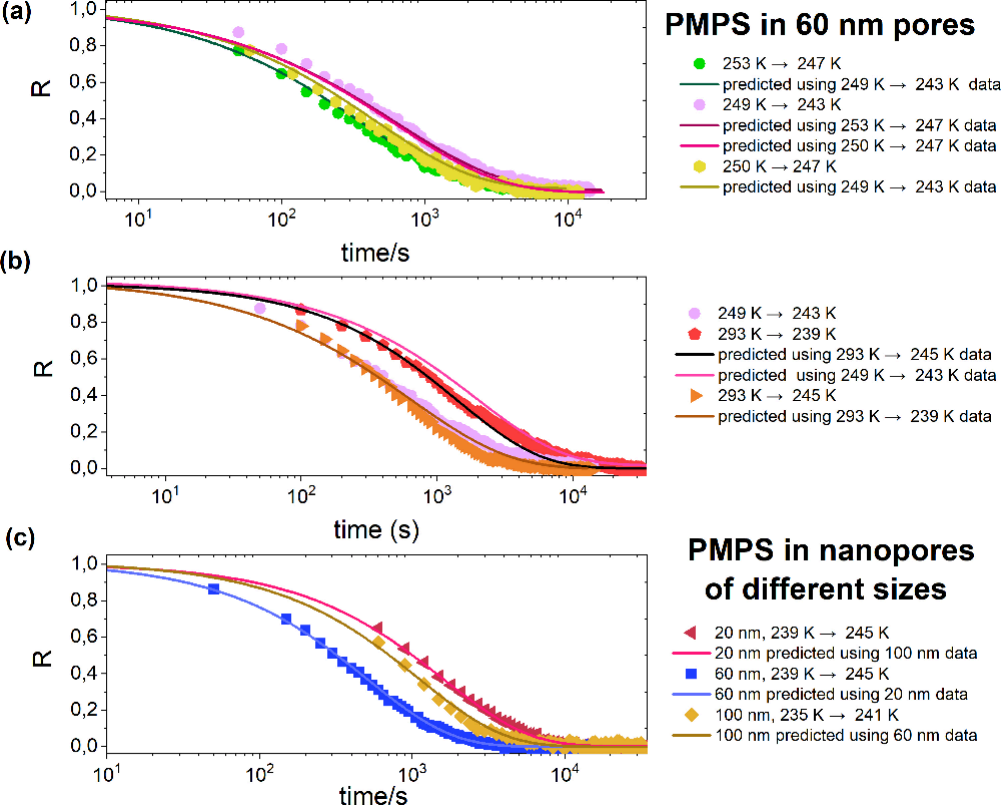
Prediction of equilibration curves in confinement based on single parameter aging tests. Experimental data measured for the tested polymer confined in alumina nanopores presented as normalized relaxation function (symbols) together with the corresponding predictions (lines) calculated based on eq 4. *R*(*t*) dependence obtained in 60 nm pores for (a) three different down jumps and (b) small (6 K) and large (>50 K) down jumps. (c) Predictions for 6 K down jumps in nanopores of different sizes.

Lastly, Figure 4c summarizes the prediction of equilibration curves in pores of various sizes. We have used three different up jumps of the same magnitude (6 K) collected in 20, 60, and 100 nm pores. The prediction of the equilibration kinetics in 20 nm pores was made based on 100 nm experimental data, while 20 nm data were used for prediction in 60 nm pores. In turn, normalized relaxation curves in 60 nm pores were used for prediction equilibration kinetics in 100 nm pores. The results demonstrate that the generalized single-parameter aging test is not limited only to equilibration phenomena in pores of the same sizes. Remarkably, it can map out equilibration phenomena at different confinement levels.

The results presented in this Letter deliver experimental evidence that the nanopore-confined system obeys single-parameter aging just like bulk glass aged just a few kelvins below *T*_g_. From the generalized single-parameter aging test, we successfully predicted equilibration kinetics for different pore sizes and jump combinations. Since the measured quantity is α-relaxation time, the clock rate is determined explicitly from the data without any assumption *a priori*. The validity of the material time concept in 2D-nanoconfinement opens up a world of new perspectives for describing equilibration phenomena of soft matter systems at the nanoscale. Along this line, it would be exciting to see how far the description in terms of single-parameter aging can be extended to other confinement dimensions, especially thin polymer films. Since equilibration phenomena in nanopore confinement share the same characteristic features as the physical aging of bulk glasses but occur for much faster relaxation times, our finding might be also very helpful for testing theoretical concepts on the generalized aging dynamics of glassy materials.

## Supplementary Material



## Notes

Information on materials, methods, raw data, additional discussion, and analysis of annealing data (PDF)
