# Supplementary material for: Predicting Equilibration Dynamics of Polymer Confined at the Nanoscale via Material Time
Source: J Phys Chem Lett. 2025 May 14;16(20):5076–81. doi: 10.1021/acs.jpclett.5c00945 (PMC12105004; doi:10.1021/acs.jpclett.5c00945)
Supplement: Supplementary file 1 [file jz5c00945_si_001.pdf]

SUPPORTING INFORMATION

Predicting Equilibration Dynamics of Polymer Confined at the Nanoscale via Material Time

Katarzyna Chat†, Ewa Sikora,‡ and Karolina Adrjanowicz‡ *

[1] *Institute of Nuclear Physics Polish Academy of Sciences, PL-31342 Krakow, Poland*

[2] *Institute of Physics, University of Silesia, 75 Pulku Piechoty 1, 41-500 Chorzow, Poland*

* Corresponding author: kadrjano@us.edu.pl

MATERIALS.

The polymer material used in this study is poly(phenylmethylsiloxane) of molecular weight M_w=2530 g/mol and polydispersity index of 1.4, labelled in the text as PMPS 2.5k. The sample was purchased from Polymer Source Inc. and used as received. The glass transition temperature of the tested polymer determined from the dielectric studies is 230 K, which perfectly agrees with the value determined based on the calorimetric investigation. In the dielectric loss spectra, we can roughly identify T_g as a temperature at which the α-relaxation time exceeds 1 second, corresponding to the α-relaxation peak maximum located at a frequency of ~ 0.16 Hz. In the glassy state, the time scale of the structural relaxation can reach geological times and cannot be approached directly.

Confinement effects were invoked by constraining PMPS 2.5k in anodized aluminum oxide (AAO) membranes of different pore sizes. AAO membranes were purchased from Inredox (USA). They are composed of uniform, non-cross-linking cylindrical pore arrays aligned perpendicular to the surface of the material and penetrating its entire thickness. The diameter of each membrane is 13 mm, while its thickness is 100 μm. In each membrane, the pore channels are aligned parallel to each other. We have used alumina templates with the following pore sizes: 20 nm, 60 nm, and 100 nm. The porosity of the membranes varies from

12 to 40 %. Before infiltration, empty membranes were dried at 473 K under vacuum for 24 hours to remove all volatile impurities from the nanopores. Infiltration of the tested polymer into the nanopores is based on capillary forces and carried out at 313 K under vacuum for two weeks. After filling, the membranes were dried using a delicate dust-free tissue. Membranes were weighed before and after infiltration. The process is assumed to be completed when the mass of the confined polymer does not change with infiltration time. Based on the membrane porosity, the density of the investigated material, and the mass of the membrane before and after infiltration, it was estimated that the nanopores filling varies from 85-95% depending on pore sizes.

METHODS.

Any viscous system initially brought out of equilibrium will slowly change its properties as it relaxes toward the equilibrium state. This process can be followed by measuring the time evolution of a certain physical quantity. Such physical quantity should be relatively strong temperature and structure-dependence so that even a small change in temperature or structure is easy to detect. Additionally, it should be measured with high precision and fast enough so that no structural relaxation occurs during the measurements. Dielectric signal satisfies all those requirements, making dielectric spectroscopy a very useful experimental technique for studying equilibration phenomena under different conditions.

All the measurements were carried out using a dielectric spectrometer Novocontrol Alpha A-analyzer. For bulk measurements of PMPS 2.5k, we have used standard plate stainless steel electrodes, separated by a Teflon spacer (10 mm diameter, 0.1 mm spacer). Dielectric studies for nanopore-confined polymer glass-formers were carried out also using plate-plate geometry, with a 100-micron distance provided by alumina membrane. Bulk and confined samples were measured as a function of temperature in the frequency range from 10^{-1} Hz to 10^{6} Hz. The temperature was controlled with stability better than 0.1 K by the Novocool system.

The time-dependent tests were carried out using the same dielectric setup. In the dielectric loss spectra, we can follow relaxation towards equilibrium by analyzing the shift of the dielectric loss curve as a function of the aging time. The dielectric permittivity upon the equilibration process in confinement must be therefore collected over a specific frequency range, not only at a single frequency. As a result, more measurement time is needed to get a single dielectric record. Generally, it makes sense to follow structural recovery via τ_α only when equilibration rates are long compared to the τ_α. This requirement is perfectly satisfied for equilibration phenomena observed in nanopore confinement, as τ_α changes after temperature jumps roughly within 10^{-3}-10^{-4} s. At the same time, the annealing times take hundreds to thousands of seconds. The real and the imaginary parts of the complex dielectric permittivity over the frequency range from 1 Hz to 10^5 Hz were monitored continuously during each annealing experiment. The time needed to collect a single spectrum did not exceed 60 seconds, which is enough to ensure that the collected data are not affected by structural changes.

Precise control over the thermal protocol is essential to achieve well-defined temperature jumps. Therefore, before performing any temperature jumps from the intermediate temperatures, we have always ensured that the nanopore-confined polymer is in the equilibrium state at a given temperature. This was confirmed by monitoring the position of α-loss peak in the dielectric spectra. In some cases, approaching equilibrium requires waiting for up to 2-3 days. Only after that do we proceed with temperature jumps of different magnitudes. For 6 K jumps, the time required to reach the desired annealing temperature does not exceed 2-3 minutes, while for larger temperature down jumps, cooling was performed with 5 K/min. Due to the limitations of our temperature regulation systems, it was not possible in this investigation to go for jumps smaller than 6 K within reasonable accuracy. For the analysis of the equilibration process, time zero was defined as the time at which the sample temperature

reaches the annealing (final) temperature with an accuracy of ± 0.1 K. Instantaneously after reaching the final annealing temperature, we started to follow equilibration kinetics tracking the changes in the position of α-relaxation process. The equilibration process was assumed to be completed when the position of α-relaxation time remains constant. For equilibration phenomena observed under nanoscale confinement, this always involves the recovery of the α-relaxation time characteristic for a bulk polymer.

When it comes to the prediction of single-parameter aging via Eq. 4 (main paper), one thing needs to be clarified. Since equilibration times in nanopore confinement are relatively fast, it is a challenge to capture both the plateau at the beginning (R=1) and the end (R=0) of the equilibration process. Therefore, to facilitate analysis, we have fitted experimental data with a stretched exponential function to reconstruct the shape of each normalized relaxation curve within the limiting range. These fitted curves - instead of the raw data - were then used for integration. In Fig. S7 we show the results of the fitting of the experimentally measured normalized relaxation functions with the use of the stretched exponential function to prove that they provide a good description of the collected equilibration curves.

ADDITIONAL RESULTS AND DISCUSSION

Introducing TN formalism and identifying material time in nanoscale confinement

The time-dependent characteristic time can be determined from the analysis of the response function $R(t)$ with the use of a relaxation function similar to stretched exponential [17, 40, 41, 42]

$$R(t) = exp\left(-\left(\frac{t}{\tau(t)}\right)^{\beta}\right), \qquad \text{(S1)}$$

where β is the nonlinear stretching exponent. In agreement with TN principles, $\tau(t)$ will also be subjected to aging and by the inverse relation linked to the aging (clock) rate. Therefore, for structural recovery in nanopore confinement, the material time, $\tilde{t}$, is determined from Eq. (1) with $\gamma(t) = 1/\ \tau(t)$. The advantage of the proposed approach is that there is no need to

perform complex mathematical modeling or extrapolations regarding equilibrium values of γ_{eq}. The analysis proceeds directly from the annealing data.

In contrast, for bulk glasses, this is far more complicated because there is no direct access to relaxation time nor the clock rate. Some extended versions of TN models assume that the reduced time can be approached using the temperature and structure shift factors. This essentially leads to time-temperature and time-aging time superpositions, where the shift factors are proportional to the structural relaxation time. [17, 40, 41, 42] The other way to address the material time concept has been developed by Glass & Time Group from Roskilde University, and it is based on first-order Taylor expansion of $X(t)$ and $\gamma(t)$ [18,19, 24]

$$\gamma(t) = \gamma_{eq}(T) \exp\left(a \frac{\Delta X(0)}{X_{eq}} R(t)\right), \qquad \text{(S2)}$$

where $a \equiv X_{eq} \frac{c_2}{c_1}$ is a dimensionless constant. Due to first-order approximation, Eq. S2 applies only to small temperature jumps. It also requires to know the equilibrium values of the clock rate γ_{eq}. Since the α-peak is outside the measurable frequency range, γ_{eq} must be estimated from the equilibrium values of τ_α using the TTS rule, or alternatively via generalized single-parameter aging tests developed by the Roskilde group.

Characteristic signatures of structural recovery response in nanoscale confinement subjected to complicated temperature-jump histories

In the original intrinsic isotherm experiment, structural recovery is followed by analyzing specific volume changes (dilatometry); here, we use structural relaxation time, which is controlled by volume effects. Before jumping to selected annealing temperatures (233 K, 237 K, 239 K, 241 K, 243 K), the sample was kept for at least a few hours at the indicated pre-annealing temperature (T_0=247 K) to ensure that it was in equilibrium. Only after that, a down jump in temperature was performed, and equilibration kinetics was followed by tracking changes in the position of the α-relaxation peak.

In asymmetry approach experiment on compares the equilibration behavior of glassy material at the same final temperature, T_{finale}, but approached following two different temperature paths of the same depth, ΔT. The two different temperature paths involve doing up and down jumps in temperature, where the 'up jump' begins at a temperature of ΔT lower than T_{finale}, while 'down jump' begins at a temperature of ΔT higher than T_{finale}. In both instances, the sample must be brought into equilibrium at the initial pre-annealing temperature, T_0 , before the temperature increment is applied.

Figure S1. Compares the time scale of equilibration processes in nanopores with α–relaxation time. As illustrated, equilibration rates in confinement are always at least 6-7 decades slower compared to τ_α.

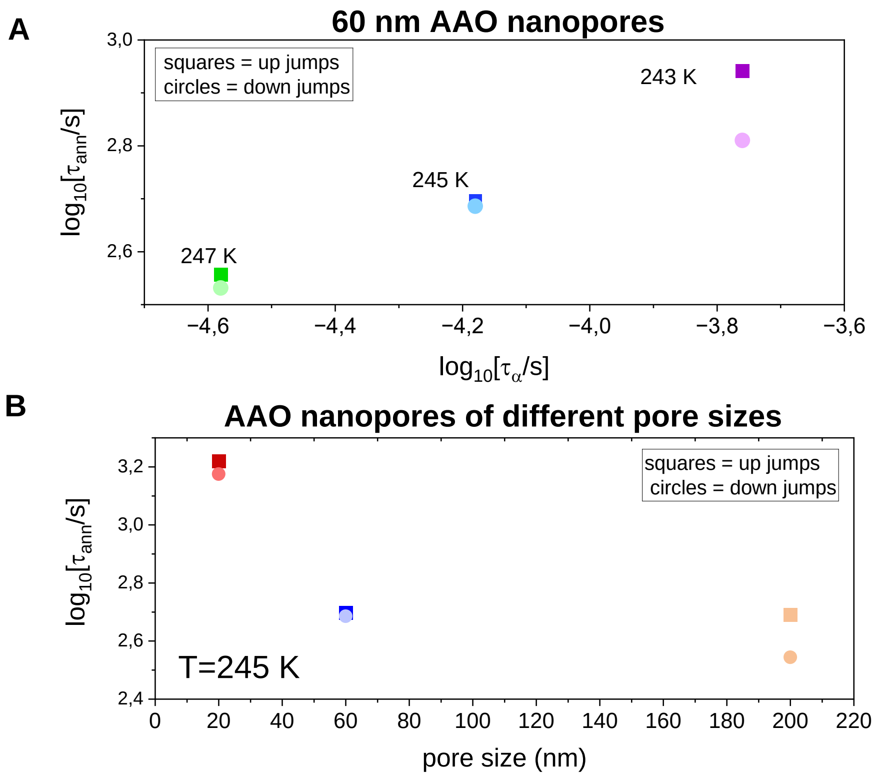

Figure S1. Differences in the time scales between τ_{ann} and τ_α (equilibrated bulk values) for PMPS 2.5k confined in AAO nanopores of (a) 60 nm pores recorded at varying temperatures, and (b) varying pore sizes but fixed equilibration temperature, T=245 K. Results shown in Fig. S1(a) corresponds to experimental data presented in Figure 2(b) of the main paper, while those presented in Fig. S1(b) to Figure 2(c) (main paper).

A test for the existence of an internal clock in nanopore confinement

According to TN formalism, the physical aging of bulk glasses can be described by a linear convolution integral that involves a material time, $\tilde{t}$. In this way, it is possible to re-establish the linearity of the aging response and simplify its mathematical description.

To test TN approach, annealing data must be presented in terms of the response function $R(t)$. In our case, this involves normalization of $\tau_\alpha(t)$ dependence according to the following formulas:

$$R(t) = \frac{\tau_\alpha(t)-\tau_{\alpha,eq}(t)}{\tau_{\alpha,t=0}-\tau_{\alpha,eq}} \quad \text{(S3)}$$

for up jumps, and

$$R(t) = \frac{\tau_{\alpha,eq}(t)-\tau_\alpha(t)}{\tau_{\alpha,eq}-\tau_{\alpha,t=0}} \quad \text{(S4)}$$

Fig. S2(a) collects $R(t)$ dependence for 6 K up and down temperature jumps to 247 K, 245 K, and 243 K in 60 nm pores, while in **Fig. S2(b)** we show the corresponding $R(\tilde{t})$ dependences. All the data collapse onto a single curve when plotted versus reduced time. This demonstrates that to describe the out-of-equilibrium phenomena of nanopore-confined glass-formers, one can use the same concept as for the physical aging of bulk glasses.

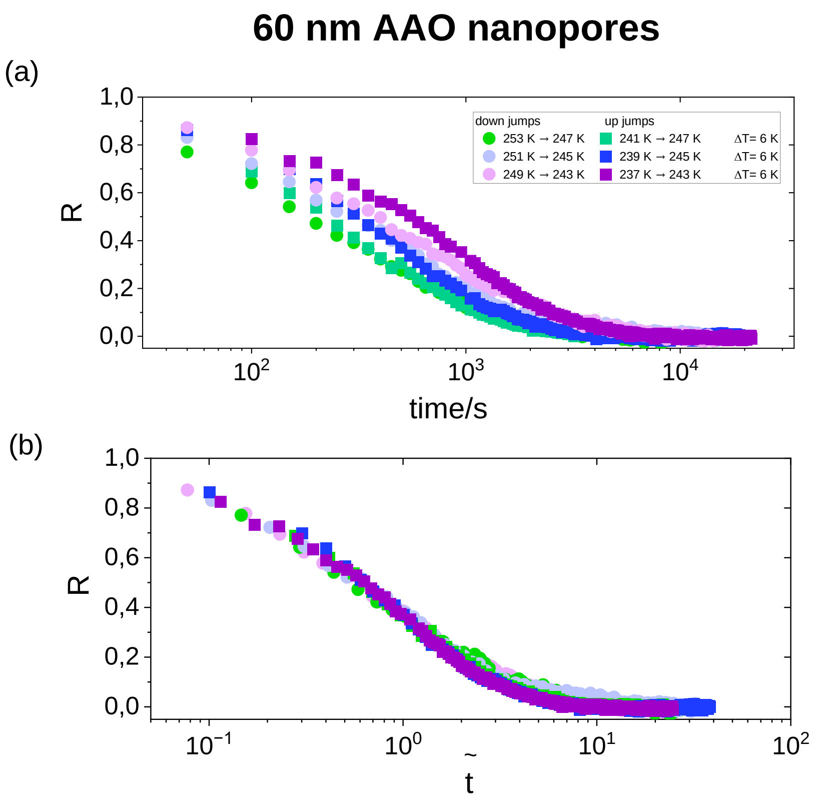

Figure S2. (a) Normalized relaxation functions for up and down temperature jumps of 6 K size as collected based on dielectric data for the tested polymer confined in 60 nm AAO nanopores. (b) The same set of curves as in (a) transformed via single-parameter aging analysis.

When two jumps end at the same temperature, $\gamma_{eq,A} = \gamma_{eq,B}$, in Eq. 4 (main paper). The position of the predicted curve is determined by $\frac{\gamma_{eq,A}}{\gamma_{eq,B}}$, while the shape of the predicted curve by $\frac{X_A(0)-X_B(0)}{X_{eq}}$. In turn, a can be found from the fitting, as a free parameter. For the tested nanopore-confined polymer, PMPS 2.5k, a=1 gives the best jump prediction. We have also noted that instead of using equilibrium values of clock rates for each pair of temperature jumps, the prediction works much better with clock rate values determined simply as $1/\tau_{ann}$.

Figure S3(a) demonstrates the prediction in 60 nm AAO nanopores for 6 K up and down jumps finishing at the same equilibration temperature, T=247 K. The up jump is from 241 K to 247 K, while the down jump is from 253 K to 247 K. Here, the up-jump data were used to predict the down jump curve and vice versa. The prediction looks quite good. In **Fig. S3(b),** we show the same R(t) dependence as in **Fig. S3(a)** but described using a function

similar to stretched exponential (in order to reconstruct the shape of the entire curve, especially as shorter equilibration times where the data are sparse). As can be seen, fitted curves provide a very good description of the corresponding experimental data.

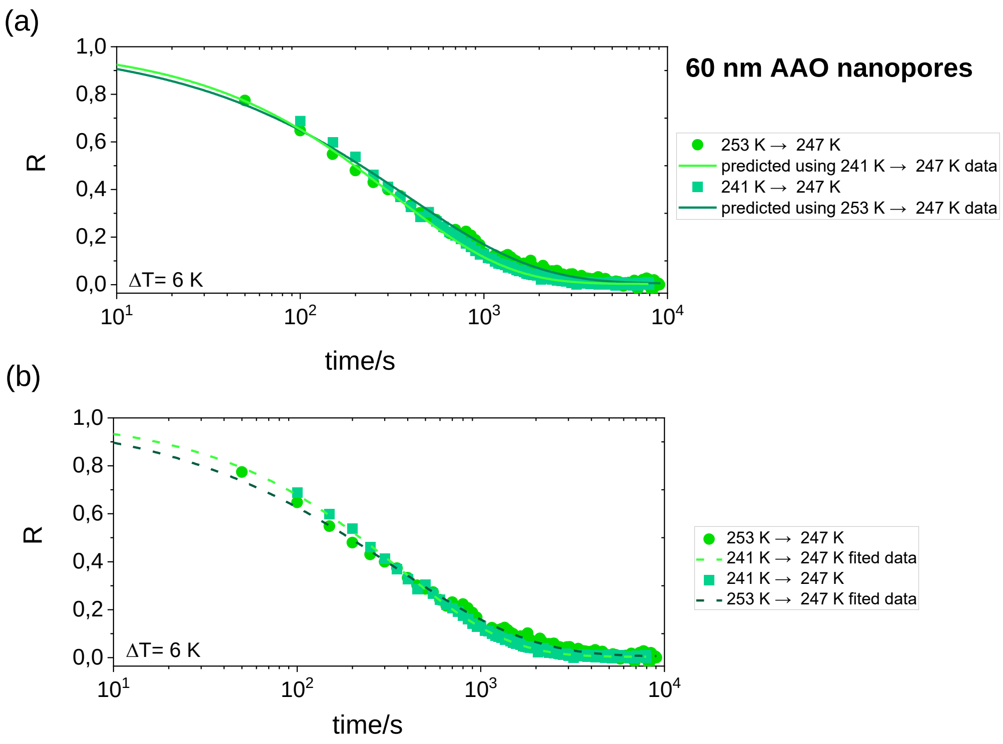

Figure S3. (a) Normalized relaxation function (symbols) together with the corresponding predictions (lines) calculated based on a single parameter aging test, Eq. 4 (a) R(t) dependences obtained for 6 K up and down in 60 nm AAO nanopores. The up and down jumps end up at the same final annealing temperature, T=247 K. (b) The same set of experimental data as in (b) but described using a function similar to stretched exponential.

RAW DATA

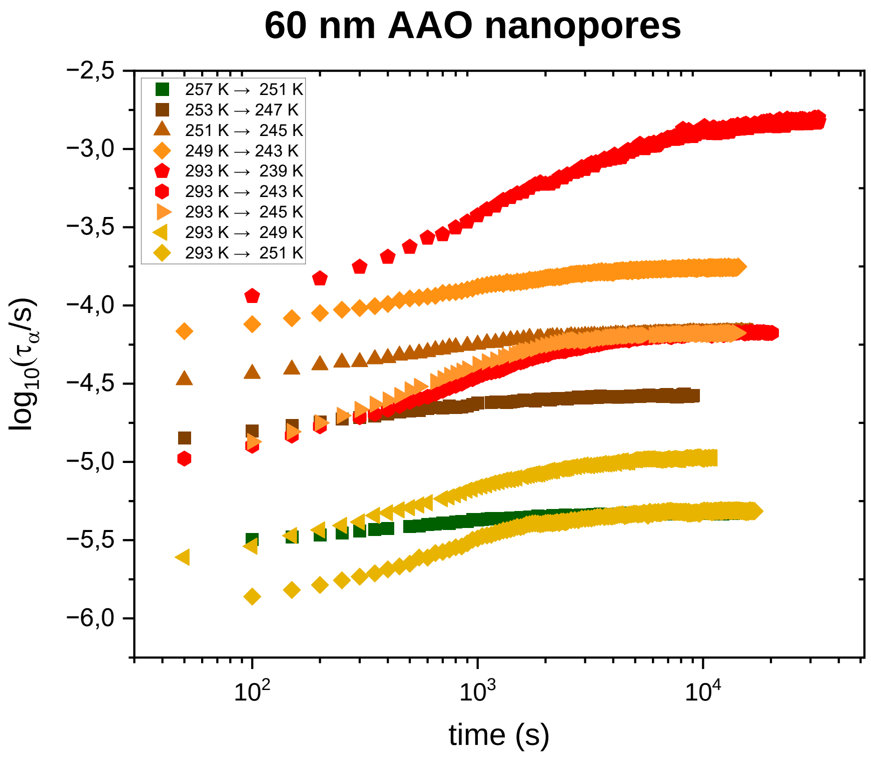

Figure S4. Temperature evolution of τ_α for PMPS 2.5k confined in 60 nm AAO nanopores before transformation to R(t) dependences as presented in Figure 3(a) (main manuscript).

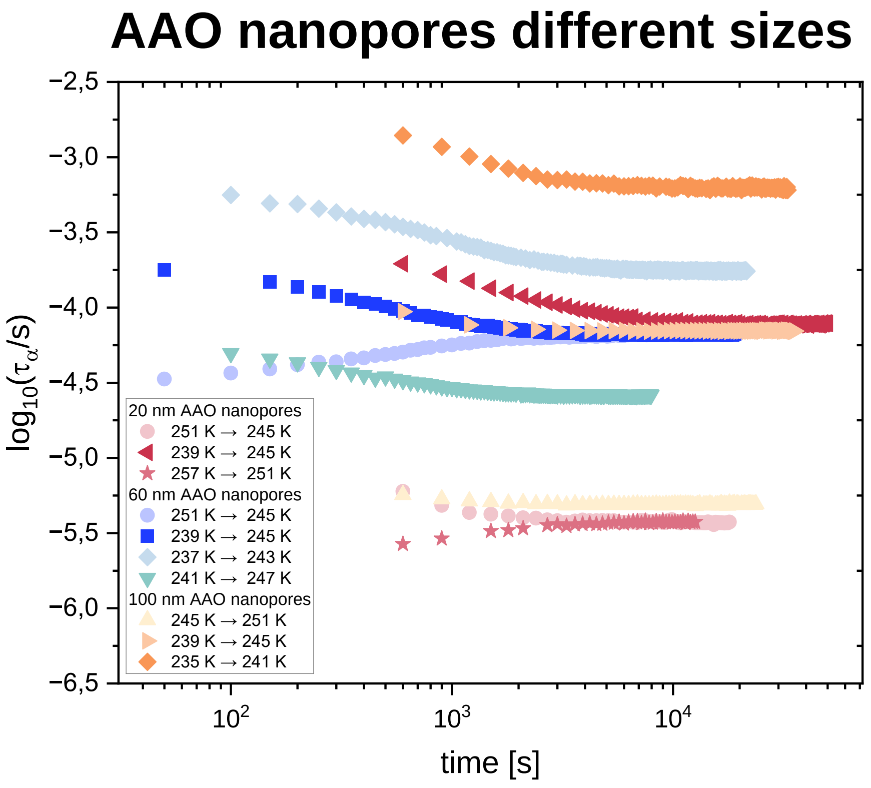

Figure S5. Temperature evolution of τ_α for PMPS 2.5k confined in 60 nm AAO nanopores before transformation to R(t) dependences as presented in Figure 3(b) (main manuscript).

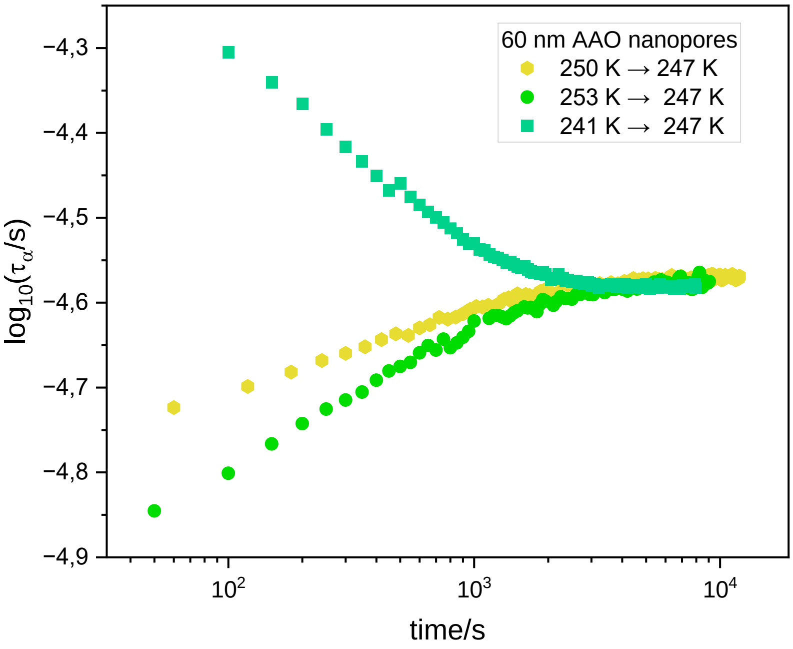

Figure S6. Temperature evolution of τ_α for PMPS 2.5k confined in 60 nm AAO nanopores before transformation to R(t) dependences as presented in Figure 4(a) (main manuscript).

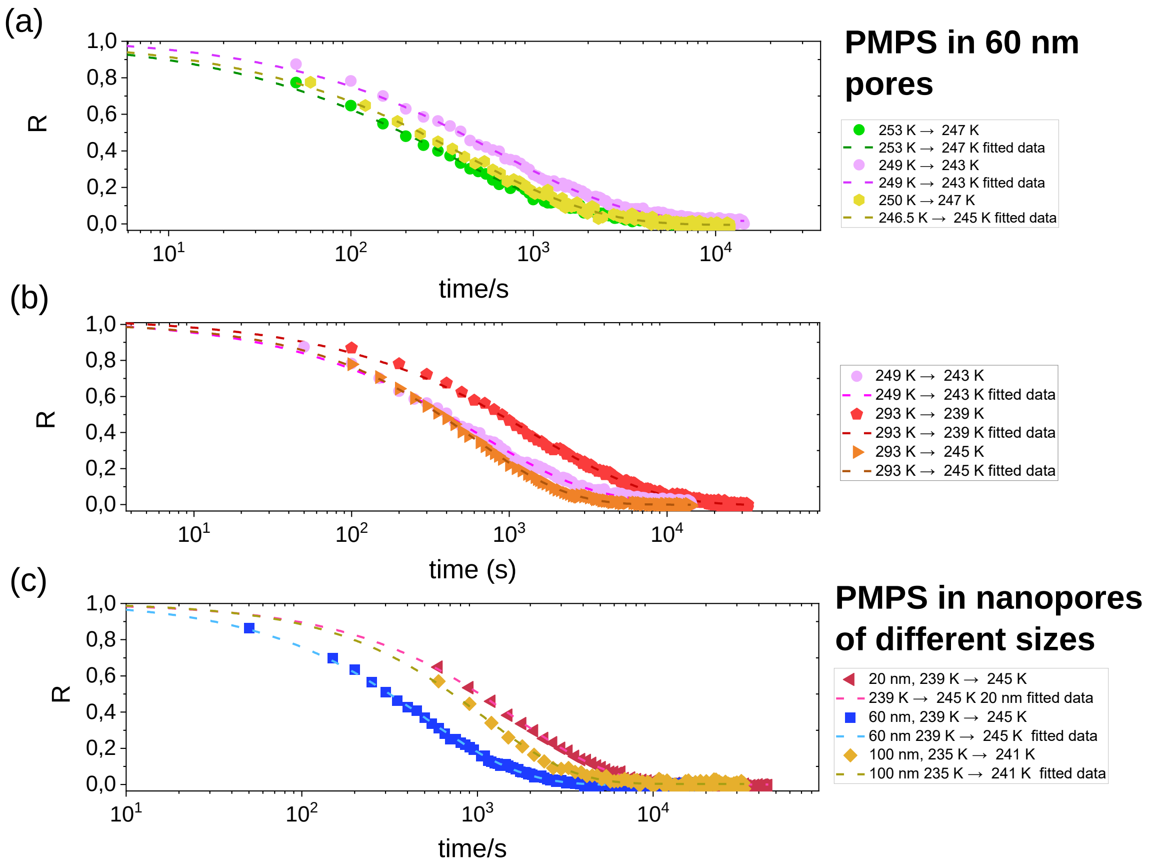

Figure S7. The same set of data as that presented in Figure 4 (main manuscript) showing normalized relaxation functions (symbols) together with the corresponding fits using a function similar to stretched exponential (dashed lines).
